# Inherited IFNAR1 Deficiency in a Child with Both Critical COVID-19 Pneumonia and Multisystem Inflammatory Syndrome

**DOI:** 10.1007/s10875-022-01215-7

**Published:** 2022-01-28

**Authors:** Hassan Abolhassani, Nils Landegren, Paul Bastard, Marie Materna, Mohammadreza Modaresi, Likun Du, Maribel Aranda-Guillén, Fabian Sardh, Fanglei Zuo, Peng Zhang, Harold Marcotte, Nico Marr, Taushif Khan, Manar Ata, Fatima Al-Ali, Remi Pescarmona, Alexandre Belot, Vivien Béziat, Qian Zhang, Jean-Laurent Casanova, Olle Kämpe, Shen-Ying Zhang, Lennart Hammarström, Qiang Pan-Hammarström

**Affiliations:** 1 Department of Biosciences and Nutrition, Karolinska Institutet, 14183 Huddinge Stockholm, Sweden; 2 Research Center for Immunodeficiencies, Pediatrics Center of Excellence, Children’s Medical Center, Tehran University of Medical Sciences, Tehran, Iran; 3 Department of Medical Biochemistry and Microbiology, Uppsala University, Uppsala, Sweden; 4 Centre for Molecular Medicine, Department of Medicine, Karolinska Institutet, Stockholm, Sweden; 5 St. Giles Laboratory of Human Genetics of Infectious Diseases, Rockefeller Branch, The Rockefeller University, New York, NY USA; 6 Laboratory of Human Genetics of Infectious Diseases, Necker Branch, Institut National de La Santé Et de La Recherche Médicale U1163, Necker Hospital for Sick Children, Paris, France; 7 University of Paris, Imagine Institute, Paris, France; 8 Division of Pediatrics Pulmonary Disease, Children’s Medical Center, Pediatrics Center of Excellence, Tehran University of Medical Sciences, Tehran, Iran; 9 Division of Clinical Immunology, Department of Laboratory Medicine, Karolinska Institute at Karolinska University Hospital Huddinge, Stockholm, Sweden; 10 Department of Human Immunology, Sidra Medicine, Doha, Qatar; 11 College of Health and Life Sciences, Hamad Bin Khalifa University, Doha, Qatar; 12 Centre International de Recherche en Infectiologie, Univ Lyon, Inserm, U1111, Université Claude Bernard, Lyon 1, Centre National de La Recherche Scientifique, UMR5308, ENS de Lyon, Lyon, France; 13 Laboratoire d’immunologie, Centre Hospitalier Lyon Sud, Hospices Civils de Lyon, Pierre-Bénite, France; 14 Paediatric Nephrology, Rheumatology, Dermatology, Hopital Femme, Mère Enfant, Hospices Civils de Lyon, Bron, France; 15 National Reference Centre for Rheumatic and Autoimmune and Systemic Diseases in Children (RAISE), Lyon, France; 16 Howard Hughes Medical Institute, New York, NY USA; 17 Department of Endocrinology, Metabolism and Diabetes, Karolinska University Hospital, Stockholm, Sweden

**Keywords:** COVID-19, critical pneumonia, multisystem inflammatory syndrome in children (MIS-C), inborn errors of immunity (IEI), primary immunodeficiency (PID), IFNAR1

## Abstract

**Background:**

Inborn errors of immunity (IEI) and autoantibodies to type I interferons (IFNs) underlie critical COVID-19 pneumonia in at least 15% of the patients, while the causes of multisystem inflammatory syndrome in children (MIS-C) remain elusive.

**Objectives:**

To detect causal genetic variants in very rare cases with concomitant critical COVID-19 pneumonia and MIS-C.

**Methods:**

Whole exome sequencing was performed, and the impact of candidate gene variants was investigated. Plasma levels of cytokines, specific antibodies against the virus, and autoantibodies against type I IFNs were also measured.

**Results:**

We report a 3-year-old child who died on day 56 of SARS-CoV-2 infection with an unusual clinical presentation, combining both critical COVID-19 pneumonia and MIS-C. We identified a large, homozygous loss-of-function deletion in *IFNAR1*, underlying autosomal recessive IFNAR1 deficiency.

**Conclusions:**

Our findings confirm that impaired type I IFN immunity can underlie critical COVID-19 pneumonia, while suggesting that it can also unexpectedly underlie concomitant MIS-C. Our report further raises the possibility that inherited or acquired dysregulation of type I IFN immunity might contribute to MIS-C in other patients.

**Supplementary Information:**

The online version contains supplementary material available at 10.1007/s10875-022-01215-7.

## Introduction

Severe acute respiratory syndrome coronavirus 2 (SARS-CoV-2) infection is asymptomatic or mild, i.e., restricted to the upper respiratory tract in about 70% of the cases [1, 2]. Moderate, non-hypoxemic pneumonia is seen in about 20% of the cases. More severe complications associated with the SARS-CoV-2 infection include severe pneumonia (about 10%), which can evolve into critical pneumonia, i.e., acute respiratory distress syndrome (ARDS, about 3%). Globally, the infection fatality rate is around 1%, but the risk of death doubles every 5 years from childhood onward, ranging from 0.001% at age 5 years to 10% at age 85 years [3, 4]. Autosomal recessive deficiency of IFNAR1 or IRF7 has been found in four unrelated adults with critical COVID-19 pneumonia [5]*.* A new patient with autosomal recessive IFNAR1 deficiency and critical COVID-19 has been recently reported [6], and a patient with TBK1 deficiency also has been identified [7]. These and other inborn errors of type I interferon (IFN) immunity had previously been reported in patients with other natural viral infections as well as in patients with live-attenuated vaccine-related viral infections [8–15]. Together with the occurrence of autoantibodies (auto-Abs) neutralizing type I IFNs in at least 15% of the cases [16, 17], these findings document an essential role of type I IFN for protective immunity to SARS-CoV-2 in the respiratory tract. These findings also led to a two-step model for COVID-19 critical pneumonia and cytokine storm, with insufficient type I IFN during the first week of infection accounting for viral spread in the lungs and other tissues, resulting in secondary inflammation during the second and third weeks of infection [5].

In contrast to COVID-19 critical pneumonia, multisystem inflammatory syndrome has been observed mostly in children (MIS-C), with MIS in adults (MIS-A) being much rarer [5, 17]. MIS-C manifests as a multiple-organ dysfunction with a delayed onset, when compared with pneumonia, usually 3–6 weeks after infection and without detectable viral shedding in the respiratory tract [18, 19]. Although MIS-C overlaps with common features of Kawasaki disease (KD, an autoimmune vasculitis), MIS-C cases are generally slightly older (8–9 years), show a higher mortality rate, a different ethnic background in affected children (more common in Asians for KD vs. Africans for MIS-C), and more prominent gastrointestinal and neurological complications [17, 20, 21]. Immunologically, MIS-C cases can be differentiated from KD using association with cytopenia, a stronger pro-inflammatory signature (but lower IL-7 and IL-8 levels), and different specificities of the detected autoantibodies [18, 22]. Unlike COVID-19 pneumonia, the etiology of MIS-C remains elusive. Intriguingly, concomitant or successive COVID-19 pneumonia and MIS-C have been reported in a few children [23]. We studied a child with this unusual presentation and hypothesized that an inborn error of immunity (IEI) may underlie the apparently concomitant presentation of both clinical manifestations in this child.

## Methods

### Study Design

The proband was identified during an evaluation of critically ill IEI patients due to COVID-19, prospectively enrolled in the Iranian national registry [24, 25]. European Society for Immunodeficiencies (ESID) criteria were used for the clinical diagnosis of the IEI [26]. Critical COVID-19 was defined as admission to the intensive care unit (ICU) due to respiratory failure, septic shock, and/or multiple organ dysfunction [27]. The diagnosis of MIS-C was defined based on the World Health Organization (WHO) criteria (Table S1) (https://www.who.int/news-room/commentaries/detail/multisystem-inflammatory-syndrome-in-children-and-adolescents-with-covid-19). The Ethics Committee of the Tehran University of Medical Science approved the study, and written informed consent was received from the parents.

### Genetic Analysis and Diagnoses

Whole-exome sequencing (WES) was performed on genomic DNA samples extracted from whole blood of the studied patient. Single nucleotide variants (SNVs), insertion/deletions (indels), and large deletions were detected using a pipeline described previously [28, 29]. The list of candidate variants was prioritized by the Combined Annotation Dependent Depletion (CADD) algorithm, and an individual gene cutoff given by using the Mutation Significance Cutoff (MSC) was considered for impact predictions [30]. The updated American College of Medical Genetics and Genomics (ACMG) criteria [31, 32] were used to re-evaluate the pathogenicity of all disease-causing gene variants, giving consideration to allele frequency in the population database, computational data, immunological results, and clinical phenotyping.

### Detection of Antibodies Specific to SARS-CoV-2 and Other Viral Infections

The presence of elevated anti-spike (anti-S1/S2) and anti-receptor binding domain (anti-RBD) levels were tested on the patients’ plasma sample as published previously [33]. Phage immunoprecipitation sequencing (PhIP-Seq) [34] was performed with an expanded version of the VirScan phage library to assess IgG antibody responses to a larger number of human-tropic viruses and selected bacterial species using species-specific cutoff values, as previously described [35–37].

### IFN Level and Cytokine Assessment

Measurement of plasma concentrations of IFN-α and other related cytokines (including IFN-γ, IL-1RA, IL-6, IL-10, IL-18, TNF, MCP-1, and soluble IL2 [sIL2]) was performed as described previously using single-molecule array (Quanterix) and simple Plex technology (ProteinSimple), respectively. Data of the proband was compared with healthy controls, pediatric COVID-19, KD, MIS-C, mild adult COVID-19, severe adult COVID-19, and toxic shock syndrome patients [38].

### IFN Autoantibody Analysis

The patient’s plasma sample was screened for autoantibodies against multiple IFNs using a bead-based protein array as published previously [39]. Briefly, recombinant human proteins were coupled to magnetic beads (MagPlex®, Luminex Corp.) containing different fluorescence markers. The AMG Activation Kit for Multiplex Microspheres (CAT#A-LMPAKMM-40) was used for coupling of target protein. The diluted samples and coupled beads were incubated together, and autoantibody binding was detected using a R-phycoerythrin-labeled goat anti-Human IgG secondary antibody (eBioScience CAT#12–4998-82) and the FlexMap 3D instrument (Luminex Corp).

### Protein Array Screening

Plasma autoantibody reactivity was studied using full-length human protein arrays (ProtoArray v5.1, PAH05251020, Thermo Fisher) [40, 41]. The patient and two healthy blood donors were investigated in the same experiment. Protein arrays were probed with plasma at a dilution of 1:2000, and otherwise following the manufacturer’s protocol. Protein arrays were first incubated with blocking buffer (PA055, Life Technologies) for 1 h, followed by 90-min incubation with plasma, and lastly, a 90-min incubation with detection antibodies: Alexa Fluor 647 goat anti-human IgG antibody (A21445, Thermo Fisher) at 1:2000 dilution and Dylight 550 goat anti-GST (#DY550011-13–001, Cayman Chemicals) at 1:10,000 dilution. The Innopsys InnoScan 1100 AL 3-channel ultra-high resolution microarray scanner was used for detection.

### Real-Time Quantitative PCR (RT-qPCR)

RNA was isolated from plasmid-transfected or un-transfected HEK293T cells with a kit according to the manufacturer’s protocol (Zymo Research). Reverse transcription was performed with random hexamers and the Superscript III reverse-strand synthesis kit according to the manufacturer’s instructions (Thermo Fisher Scientific). RT-qPCR was performed with Applied Biosystems Taqman assays for IFNAR1 and the β glucuronidase housekeeping gene for normalization. Results are expressed according to the ΔΔCt method, as described by the kit manufacturer.

### IFNAR1 Overexpression by Plasmid Transfection

An *IFNAR1* containing plasmid was used to transfect HEK293T cells for overexpression of the wild type (WT) or the mutants by incubation for 36 h in the presence of X-tremeGene 9 transfection reagent (Sigma-Aldrich). One microgram of plasmid was used to transfect 0.5 × 10^6^ cells [8].

### Western Blotting

HEK293T cells were transfected for 36 h with plasmids (pGEMT cloning vector, Promega) containing WT or the mutated *IFNAR1*. Cells were lysed in NP-40 lysis buffer (280 mM NaCl, 50 mM Tris, pH 8, 0.2 mM EDTA, 2 mM EGTA, 10% glycerol, and 0.5% NP-40) supplemented with 1 mM dithiothreitol, PhosSTOP (Roche), and complete protease inhibitor cocktail (Roche). The protein lysate was subjected to SDS-PAGE, and the bands obtained were transferred to a nitrocellulose membrane. For measuring the protein overproduced following transfection, we used a polyclonal anti-IFNAR1 antibody recognizing the C-terminus of IFNAR1 (ab45172, Abcam) and anti-GAPDH (as loading control gene, sc-47724, Santa Cruz Biotechnology). The membrane was incubated overnight at 4 °C with the primary antibodies. For detection of the protein overproduced following transfection, we used a secondary antibody from Li-COR (IRDye 800CW anti-mouse and IRDye 680RD for anti-rabbit). Membranes were then read using Li-COR.

### Flow Cytometry

For measurement of the cell surface expression of IFNAR1, HEK293T cells were transfected for 36 h with WT or mutant *IFNAR1,* and both were subsequently surface-stained with purified mouse anti-IFNAR1 (AA3 custom antibody, PBL Assay Science). Cells stained with AA3 were then washed once with PBS and incubated with a biotinylated rat anti-mouse secondary antibody (Thermo Fisher Scientific) for 30 min, before being washed once with PBS and incubated for 30 min with PE-conjugated streptavidin (Thermo Fisher Scientific). The cells were then washed twice with PBS and analyzed by flow cytometry. Data were acquired on a Gallios flow cytometer (Beckman Coulter), and the results were analyzed with the FlowJo software (Tree Star).

### Generation of IFNAR1-Deficient HEK293T Cells

IFNAR1-deficient HEK293T cells were generated with the CRISPR/Cas9 system. Guide RNAs were designed with the Benchling design tool and inserted into lentiCRISPR v2, which was a gift from Feng Zhang (Broad Institute, Cambridge, MA; plasmid 52,961; Addgene). The three guide RNAs were designed to bind and cut at different places in the *IFNAR1* gene, one in exon 3 (forward: 59-CACCGCATATGAAATACCAAACACG-39; reverse: 5′-AAACCGTGTTTGGTATTTCATATGC-3′), one in exon 4 (forward: 5′-CACCGCATTGCTGTATACAATCATG-3′; reverse: 5′-AAACCATGATTGTATACAGCAATGC-3′), and one in exon 5 (forward: 5′-CACCGTGAGTGGAGAAGCACACACG-3′; reverse: 5′-AAACCGTGTGTGCTTCTCCACTCAC-3′). Using X-tremeGENE 9 DNA Transfection Reagent (Roche), we transiently transfected WT HEK293T cells with the resulting plasmids and cultured them for 7 days before sorting IFNAR1-deficient cells by flow cytometry after staining with an Ab against IFNAR1(AA3, custom antibody). The three resulting cell lines were subsequently tested to check for a lack of IFNAR1 expression at the cell surface and were used in the luciferase assays.

### Luciferase Reporter Assays for IFNAR1 Functional Testing

IFNAR1^−/−^ HEK293T cells generated by the CRISPR/Cas9 system were transfected with the indicated expression plasmids, firefly luciferase plasmids, under the control of WT or mutant IFNAR1 variants or human IFN-sensitive response element (ISRE) promoters in the pGL4.45 backbone, and a constitutively expressing Renilla luciferase plasmid for normalization (pRL-SV40). Cells were transfected in the presence of the X-tremeGene 9 transfection reagent (Sigma-Aldrich) for 36 h. Cells were then either left unstimulated or were stimulated with IFN-α2 (Milteny Biotec, ref. number 130–108-984), IFN-ω (Merck, ref. number SRP3061), at 10 ng/mL or 100 pg/mL, or IFN-β (Milteny Biotech, ref. number: 130–107-888) at 10 ng/mL, for 16 h at 37 °C. Each sample was tested three times for each cytokine. Finally, cells were lysed for 20 min at room temperature, and luciferase levels were measured with the Dual-Luciferase® Reporter 1000 assay system (Promega, ref. number E1980), according to the manufacturer’s protocol. Luminescence intensity was measured with a VICTOR-X Multilabel Plate Reader (PerkinElmer Life Sciences, USA). *Firefly* luciferase activity values were normalized against *Renilla* luciferase activity values. These values were then normalized against the median induction level for non-neutralizing samples and expressed as a percentage. Samples were considered neutralizing if luciferase induction, normalized against *Renilla* luciferase activity, was below 15% of the median values for controls tested the same day.

### Detection and Functional Evaluation of Anti-Cytokine Autoantibodies

Cytokines, recombinant human (rh) IFN-α2 (Milteny Biotec, ref. number 130–108-984) or rhIFN-ω (Merck, ref. number SRP3061), were first biotinylated with EZ-Link Sulfo-NHS-LC-Biotin (Thermo Fisher Scientific, cat. number A39257), according to the manufacturer’s instructions, with a biotin-to-protein molar ratio of 1:12. The detection reagent contained a secondary antibody (Alexa Fluor 647 goat anti-human IgG, Thermo Fisher Scientific, ref. number A21445) diluted in Rexip F (Gyros Protein Technologies, ref. number P0004825; 1:500 dilution of the 2 mg/mL stock to yield a final concentration of 4 µg/mL). Buffer PBS-T 0.01% and Gyros Wash buffer (Gyros Protein Technologies, ref. number P0020087) were prepared according to the manufacturer’s instructions. Plasma samples were then diluted 1:100 in PBS-T 0.01% and tested with the Bioaffy 1000 CD (Gyros Protein Technologies, ref. number P0004253) and the Gyrolab X-Pand (Gyros Protein Technologies, ref. number P0020520). Cleaning cycles were performed in 20% ethanol.

The blocking/neutralizing activity of anti-IFN-α2 and anti-IFN-ω auto-Abs was further determined with a reporter luciferase activity. Briefly, HEK293T cells were transfected with a plasmid containing the *Firefly* luciferase gene under the control of the human *ISRE* promoter in the pGL4.45 backbone and a plasmid constitutively expressing *Renilla* luciferase for normalization (pRL-SV40). Cells were transfected in the presence of the X-tremeGene9 transfection reagent (Sigma-Aldrich, ref. number 6365779001) for 24 h. Cells in Dulbecco’s modified Eagle medium (DMEM, Thermo Fisher Scientific) supplemented with 2% fetal calf serum (FCS) and 10% healthy control or patient plasma (after inactivation at 56 °C, for 20 min) were either left unstimulated or were stimulated with IFN-α2 (Milteny Biotec, ref. number 130–108-984), IFN-ω (Merck, ref. number SRP3061), at 10 ng/mL or 100 pg/mL, or IFN-β (Milteny Biotech, ref. number: 130–107-888) at 10 ng/mL, for 16 h at 37 °C. Each sample was tested once for each cytokine and dose. Finally, luciferase levels were measured as described above.

## Results

### Patient Characteristics

**Fig. 1:**
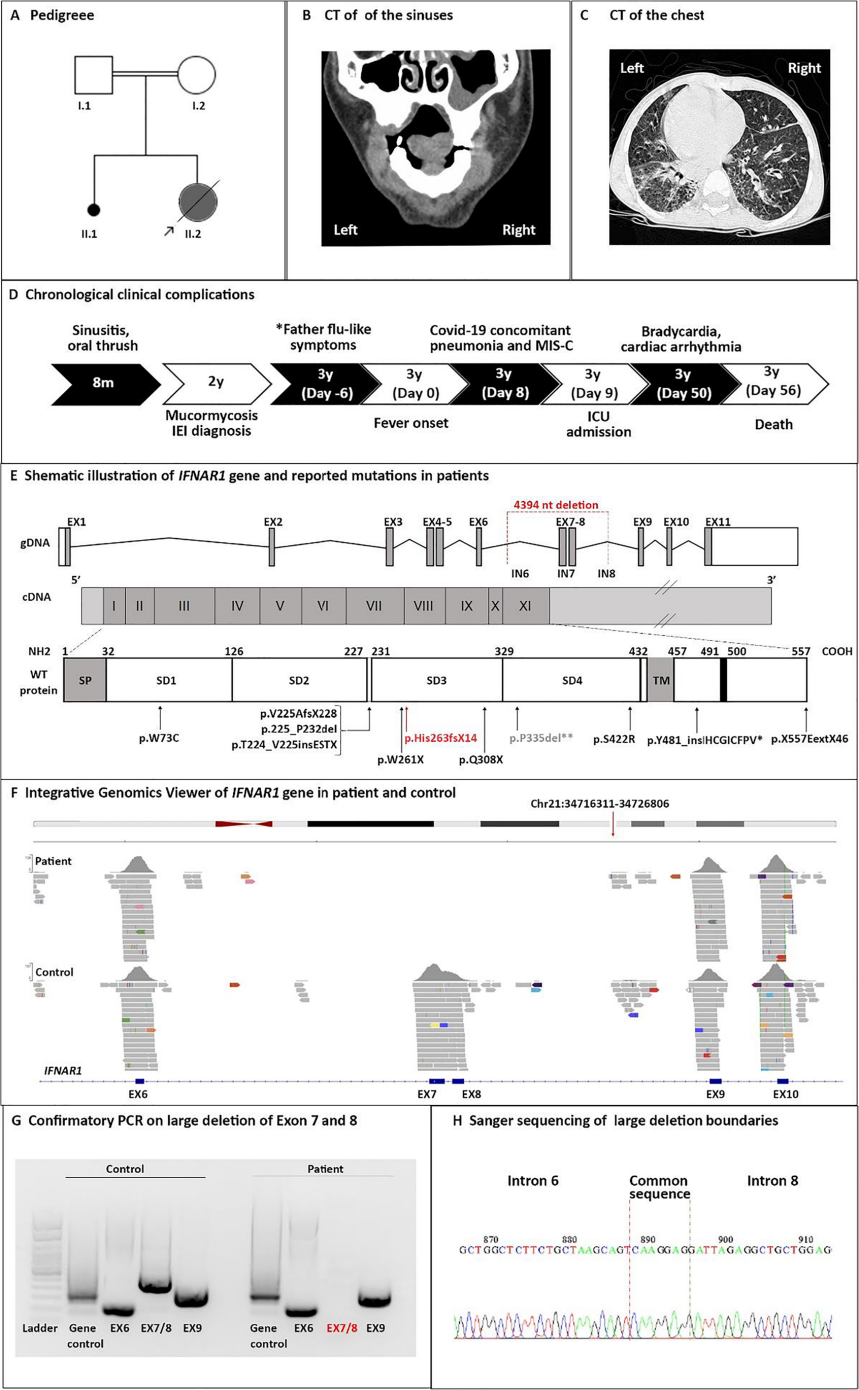
Clinical and genetic evaluation of a patient with IFNAR1 deficiency associated with both critical COVID-19 pneumonia and MIS-C. Panel **A** shows the pedigree of the index patient; Panel **B** shows computed tomography (CT) scan revealing right sinus affected by mucormycosis at age 2; Panel **C** shows chest CT scan day 8 after the onset of the disease, with ground-glass opacification and dense consolidation on air bronchograms; Panel **D** shows chronological clinical complications in a patient; Panel **E** depicts a schematic illustration of the localization of the large deletion identified in this report and mutations identified in previously reported cases with IFNAR1 deficiency. IEI inborn errors of immunity. Panels **F–G** represent the confirmation of large deletion in the index patient by integrative genomics viewer of the whole-exome sequencing, PCR, and Sanger sequencing

The patient was a 3-year-old girl, born through cesarean section to first-cousin consanguineous healthy parents, and living in Iran (Fig. 1A). There was no family history of immune-related disease, but there was one previous stillbirth sibling at 4 months of pregnancy. The patient was thriving normally with no adverse reaction to live attenuated vaccines, including Bacillus Calmette–Guérin (BCG) at birth, oral polio vaccine (OPV) at birth—2, 4, 6, and 18 months, and measles, mumps, and rubella (MMR) at 12 and 18 months. At the age of 8 months, he began to suffer from severe chronic sinusitis (low-grade symptoms and signs that persisted for longer than 12 weeks despite antibiotics and nasal steroids) and oral thrush. Shortly afterwards, at age 2 years, she developed severe mucormycosis of the nose and paranasal sinuses (without associated chronic lung disease, Fig. 1B), which improved after amphotericin B treatment for 35 days. The conventional tests failed to show any evidence of common fungal species including Candida or Aspergillus species. An immunological evaluation was performed at this age, which, however, showed largely normal T and B cell parameters (Table 1). Secondary immunodeficiency due to HIV was also excluded. The patient was considered to have an unclassified defect in innate immunity and underwent routine follow-up every 3 months.

**Table 1:** Immunologic profile of a patient with both critical COVID-19 pneumonia and MIS-C

Parameter	Before SARS-CoV-2 infection (2 years)	After SARS-CoV-2 infection (3 years)	Normal range
IgG (mg/dL)	1490	1604	700–1600
IgA (mg/dL)	220	185	19–220
IgM (mg/dL)	270	130	55–210
Hemoglobin (g/dL)	11.0	10.3	11.5–15.5
Platelets (cell × 10^9^/L)	250	548	200–500
WBC (cell/μL)	14,200	14,700	5000–15,000
Monocytes (cell/μL)	420	470	200–1000
Lymphocytes (cell/μL)	5680	5320	2000–10,000
CD3^+^ T cells (%)	60	64	30–78
CD4^+^ T helper cells (%)	40	42	22–58
CD8^+^ T cytotoxic cells (%)	18	20	10–37
CD19 + B cells (%)	23	21	3–14
NBT test (%)	100%	NI	90–100
Lymphocyte transformation test	Normal	Normal	Normal
C3 (mg/dL)	105	175	90–180
C4 (mg/dL)	20	36	15–40
CH50 (%)	91	95	75–125
HIV DNA PCR	Negative	NI	Negative
ALT (U/L)	32	158	0–40
AST (U/L)	28	163	0–40
GGT (U/L)	NI	76	0–20
CRP (mg/L)	15	229	0–14
ESR (mm/h)	21	29	0–15
Ferritin (ng/mL)	NI	198	10–140
CK (U/L)	53	5320	40–200
cTnI (ng/mL)	NI	0.82	0–0.5
D-dimers (mg/L)	NI	1.1	0.5–2.0

*WBC white blood cells, ALT alanine aminotransferase, AST aspartate transaminase, CRP C-reactive protein test, CK creatine kinase, cTnI cardiac troponin I, GGT gamma- glutamyl transferase, NBT nitro blue tetrazolium, NI not indicated, CH50 50% haemolytic complement activity, ESR erythrocyte sedimentation rate*

At age 3 years, she presented in late September 2020 with an 8-day history of low-grade fever (37.5–38.0 °C), severe dyspnea, arthralgia, myalgia, acute gastrointestinal symptoms (nausea, diarrhea, ileus, and feeding intolerance), bilateral non-purulent conjunctivitis, and maculopapular rash. Her mother was symptom-free, but her father had flu-like symptoms 2 weeks before the hospital admission of the child. However, at the time of hospitalization of the patient, both parents were negative for SARS-CoV-2 based on a RT-PCR test. On physical examination at admission, the child was irritable and febrile (39.1 °C), had a coarse breath sound and shortness of breath with fine crackles in pulmonary auscultation, hypotension, strawberry tongue as well as lymphadenopathy. Full blood investigation showed anemia, leukocytosis, increased platelet counts, increased transaminases, increased acute-phase reactants, and pyuria. Electrocardiogram showed raised S-T segments, and echocardiography demonstrated myocardial edema in the basal inferoseptal crypt and a dilated right coronary artery (0.29 cm, z-score of 2.5 indicating dilation only based on the American Heart Association classification [42], left ventricular ejection fraction of 63.1%). Blood gases demonstrated a metabolic acidosis, and elevated levels of cardiac biomarkers (Table 1) reflected myocardial injury. Initial supportive cardiovascular treatment started with supplemental oxygen, positive pressure ventilation, and intravenous inotropic support as well as angiotensin-converting enzyme inhibitors and amiodarone in advanced stages. Chest computed tomography revealed ground-glass opacification and dense consolidation air bronchograms (Fig. 1C).

RT-PCR test for SARS-CoV-2 on a nasopharyngeal swab at day 8 after the onset of fever was positive. Based on the clinical manifestations, a diagnosis of pneumonia and MIS-C due to SARS-CoV-2 infection was made. Despite initiation of immunomodulatory dose of intravenous immunoglobulin (IVIg, 1 g/kg for 2 days), the condition of the patient deteriorated (with sudden oxygen desaturation to 80%), and she was transferred to the Pediatric Intensive Care Unit (PICU) at day 2 of the hospitalization. Augmented immunomodulatory treatment was continued with aspirin (50 mg/kg/day) and prednisolone (2 mg/kg/day) and in the last stage together with infliximab (5 mg/kg). After 2 days in the PICU, he was intubated and put on mechanical ventilation. Further investigation of the patient during the 4 weeks of hospitalization in the PICU indicated hepatosplenomegaly and mild peritoneal effusion on abdominal ultrasound. Treatment including intravenous antibiotics, IVIg, acetaminophen, ibuprofen, clopidogrel was ineffective, and she suffered from persistent fever, bradycardia, cardiac arrhythmia, and respiratory distress and died at day 48 of hospitalization due to respiratory and cardiac failures (acute left ventricular failure complicated by refractory ventricular tachyarrhythmia, Fig. 1D and Fig. S1).

### Autosomal Recessive IFNAR1 Deficiency

Due to the severity of the disease and previous history of a suggested inborn error of innate immunity, a sample was collected for genetic evaluation and specific immune responses on the second day of PICU admission. WES was performed and analyzed. Rare non-synonymous variants identified in homozygous and heterozygous states in the proband are listed in Tables S2 and S3, respectively. A homozygous large deletion (4394 bp, chr21: 34,719,302–34,723,696) in *IFNAR1*, encompassing exons 7 and 8, was identified and validated by PCR and Sanger sequencing (Fig. 1E–H, Table S4–5, Fig. S2–3). This alteration was predicted to create a frameshift and premature stop codon at the 5′ end of *IFNAR1* cDNA, resulting in the truncation of the IFNAR1 protein before its transmembrane domain (p.H263fs14*, NM_000629), thus highly likely a loss-of-function.

**Fig. 2:**
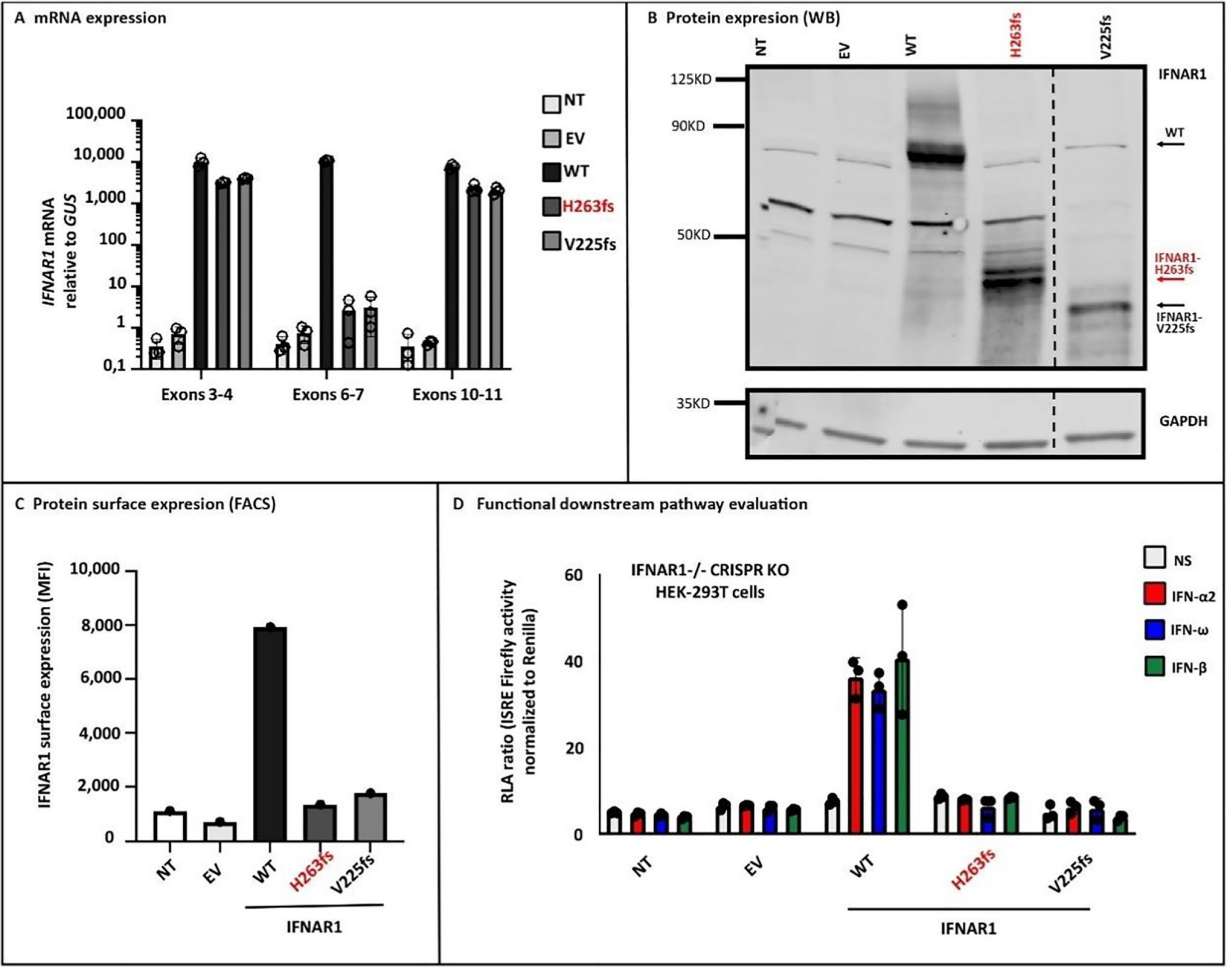
The patient’s *IFNAR1* variant (H263fs*) is not expressed at the cell surface and is a loss-of-function variant. Overexpression experiments that were performed in HEK293T cells showed normal mRNA expression except when using a probe spanning exons 6–7 (Panel **A**), and truncated protein expression detected by western blotting (WB, Panel **B**) and a very low level of cell surface IFNAR1 expression by fluorescence-activated cell sorting (FACS, Panel C) in the H263fs*mutant compared to the wild type (WT) protein. Panel D depicts IFN-sensitive response element (ISRE) activity induction upon stimulations with IFN-α2, IFN-ω, or IFN-β in IFNAR1^−/−^ cells where different WT or mutant IFNAR1 plasmids were transiently transfected and the H263fs* mutant does rescue the response in contrast to the WT variant. NT nontransfected cell lines, EV empty vector, MFI mean fluorescent intensity, KO knockout

No cells from the patient were available for functional studies. We thus constructed the patient’s mutant (H263fs* variant) in an *IFNAR1* plasmid and tested its expression and function in vitro. Following the transient transfection of cells with plasmids containing the WT or mutant *IFNAR1* cDNA, similar levels of *IFNAR1* mRNA were detected for the WT and mutant by RT-qPCR with probes covering exons 3–4 or 10–11 of IFNAR1, whereas no IFNAR1 cDNA was detected for the mutant IFNAR1 when a probe covering exons 6–7 of IFNAR1 was used, consistent with the deletion in exon 7–8 (Fig. 2A). Western blot analysis of these cell extracts with an antibody specific for the N-terminal region of IFNAR1 revealed a mutant IFNAR1 band, at a lower molecular weight than the WT (Fig. 2B). We then analyzed the cell surface expression by FACS and found that the cell-surface IFNAR1 expression was almost undetectable when compared to WT (Fig. 2C), similar to another reported pathogenic truncated protein, V225fs* [14]. Finally, we used the WT or mutant IFNAR1 cDNA to transfect IFNAR1^−/−^ HEK293T cells, which we created by CRISPR/Cas9-mediated gene editing. Upon stimulation with IFN-α2, IFN-ω, or IFN-β and transfection with a reporter gene, the cells expressing WT IFNAR1 displayed luciferase activity, unlike those expressing mutant H263fs* IFNAR1, or the previously reported loss-of-function variant V225fs* due to lack of IFN-sensitive response element (ISRE) activation (Fig. 2D). Thus, the proband mutation H263fs* is loss-of-expression and loss-of-function, suggesting that the patient had an autosomal recessive complete IFNAR1 deficiency.

### Specific Virus Antibody Responses and Auto-Antibody Profiling

No specific antibodies against the spike (S) or the receptor binding domain (RBD) of the S protein of SARS-CoV-2 were detected in the blood at day 2 of hospitalization using an enzyme-linked immunosorbent assay (ELISA) method [33]. We next performed PhIP-Seq to investigate the historical humoral immune response against other viral and a smaller number of bacterial infections in the patient and observed presence of specific IgG antibodies toward Herpesviridae families (including Epstein-Barr virus and Cytomegalovirus), human respiratory syncytial virus, and cross-reactivity with Middle East respiratory syndrome coronavirus (MERS-CoV) but not with SARS-CoV antigens (Fig. S4). Of note, cross-reactive antibodies to MERS-CoV were similar to our previous observation in adult patients with severe COVID-19 [37]. We also measured selected cytokines in the plasma sample from the patient and observed higher levels of TNF and sIL2 as compared to the healthy controls, a pattern that is distinct from patients with typical MIS-C, KD, severe adult COVID-19, or toxic shock syndrome (Fig. S5). Finally, we screened our patient for plasma autoantibodies against multiple IFN species and more than 9000 additional human proteins. No autoantibodies against type I IFNs or IL17 were detected (nor any blocking activity of anti-IFN-α2 and anti-IFN-ω auto-Abs, Table S6–7, Fig. S6), whereas several other putative autoantibody targets were identified, including KLK7 (kallikrein 7), PLAUR (urokinase plasminogen activator surface receptor), and S100A1 (S100 calcium binding protein A1, Fig. S7).

## Discussion

We report a child who died of critical COVID-19 pneumonia and concomitant MIS-C due to autosomal recessive, complete IFNAR1 deficiency. Previously, only nine patients have been reported with autosomal recessive IFNAR1 deficiency worldwide since its first observations in 2019 [5, 6, 8, 14, 43]. Adverse reactions to MMR (*n* = 2) [14, 43] and yellow fever vaccines (*n* = 1) [14], as well as herpes simplex encephalitis and other viral infections (*n* = 2) [8], cytomegalovirus viremia (*n* = 1) [44], and life-threatening COVID-19 (*n* = 3) [28, 35] have been reported in these cases (Fig. 1E, Table 2). Another patient with an autosomal dominant form of IFNAR1 deficiency and critical adulthood COVID-19 pneumonia has also been reported (Fig. 1E, Table 2) [5]. Individuals with autosomal recessive IFNAR1 deficiency described thus far, including our patient, were aged 6 months to 38 years and, surprisingly, had suffered from relatively few viral infections, consistent with the clinical features of patients with IFNAR2 deficiency [45]. Although the patients with IFNAR1 deficiency can be otherwise healthy until adulthood, they may still suffer from a high mortality in an episode of viral infection (3/10 dead due to viral infection, 30%). Even patients unresponsive to both type I and III IFNs, due to mutations in *STAT2* or *IRF9*, have a narrow infectious phenotype [12, 13, 46]. Our result confirms and extends the finding that type I IFN signaling can be redundant in host defense against at least some common viruses, but defects in this pathway may also underlie life-threatening viral infections and associated complications. Importantly, our patient is the fourth patient with critical COVID-19 pneumonia due to autosomal recessive IFNAR1 deficiency, confirming that type I IFN is essential for protective immunity against SARS-CoV-2 in the respiratory tract [33].

**Table 2:** Summary of reported patients with IFNAR1 deficiency

Gender	Age	Mortality	Inheritance	Zygosity	mRNA change	Amino acid change	Natural viral infection	Vaccine side effects	References
Male	9 years	-	AR	Hom	c.674–2 A > G	p.V225fs*	-	MMR	Hernandez et al
Female	12 years	-	AR	Comp Het	c.674–1 G > A	p.V225fs*	-	YFV	Hernandez et al
					c.783 G > A	p.W261*			
Female	6 months	Dead	AR	Hom	Large del exon 11	p.Y481fs*	HSV	*	Bastard et al
Male	13 months	-	AR	Hom	Large del exon 11	p.Y481fs*	**	-	Bastard et al
Male	15 months	Dead	AR	Hom	c.992 C > T	p.Q308*	EBV	MMR	Gothe et al
Male	2 months	-	AR	Hom	c.1671_1821 del	p.*557E ext46	CMV	-	Hoyos-Bachiloglu et al
Male	38 years	-	AR	Hom	c.219 G > C	p.W73C	COVID-19	-	Zhang et al
Male	26 years	-	AR	Hom	c.1264 A > G	p.S422R	COVID-19	-	Zhang et al
Male	13 years	-	AR	Hom	c.674-2A > G	p.V225fs*	COVID-19, influenza	MMR	Khanmohammadi et al
Female	23 years	-	AD	Het	c.1000 delCCT	p.P335del	COVID-19	-	Zhang et al
Female	3 years	Dead	AR	Hom	Large del exons 7–8	p.H263fs*	COVID-19	-	This study

*AR autosomal recessive, AD autosomal dominant, Hom homozygous, Het heterozygous, Comp Het compound heterozygous, MMR measles, mumps, and rubella vaccine, YFV yellow fever vaccine, HSV herpes virus infection, EBV Epstein-Barr virus, CMV cytomegalovirus*

^*^The patient had one night of fever after MMR vaccine and atypical Kawasaki disease, without typical cardiac involvement

^**^Viral serological data showed him to be positive for antibodies against mumps, HSV-1, human herpes viruses 4, 5, and 6, rhinovirus, adenovirus, enterovirus B, varicella zoster virus (VZV), cytomegalovirus, EBV, measles virus, mumps virus, hepatitis A virus, influenza A virus, and respiratory syncytial virus

Although our patient did not present any side effects to live attenuated vaccines, a severe fungal infection (mucormycosis) developed at 2 years of age, prior to the SARS-CoV2 pandemic. One possibility is that another IEI is underlying this unusual phenotype. However, this patient did not carry any variants in *CARD9*, *IL17RA*, *L17RC*, *IL17F*, *STAT1*, *DOCK8*, *MALT1*, *or TRAF3IP2* genes that can explain the susceptibility to a severe and invasive fungal infection. Alternatively, mucormycosis in this patient was secondary to an undocumented viral infection due to the IFNAR1 deficiency. This is supported by the recent surge of reported mucormycosis cases associated with COVID-19 from India, mostly in immunocompromised or diabetic subjects [47, 48].

The MIS-C observed in this child is a novel phenotype associated with IFNAR1 deficiency. Intriguingly, progressive hemophagocytosis after MMR vaccine or cytomegalovirus infections was noted in two of the previously reported cases [43, 44], as well as in patients with other defects in the type I IFN pathway including STAT1 [49], STAT2 [50, 51], and IFNAR2 [9, 52]. Despite exaggerated inflammation, our patient did not fulfill the diagnostic score of hemophagocytosis (Hscore [53]: 106). Moreover, atypical KD without typical cardiac involvement was noted in another IFNAR1-deficient patient leading to a severe course of disease (30-day hospitalization, recovered after IVIg) [8]. These related observations suggest that correctly tuned type I IFN response is required for a proper regulation of proinflammatory cytokines, a notion which fits with the recent observation of overzealous cytotoxic responses in MIS-C patients [54]. A direct invasive potential SARS-CoV-2 infection of endothelial cells has previously been proposed in MIS-C autopsy studies [55]. However, autopsy was not consented in the current patient, but it is possible that IFNAR1 deficiency might increase the chance of invasive infection of heart endothelial cells. Of note, MIS-C overlapping with acute COVID-19 pneumonia in a US cohort comprised 30% of the whole MIS-C cohort [23]. Many children with this overlapping phenotype presented with respiratory involvement, including cough, shortness of breath, pneumonia, and occasionally ARDS. Most of these children had positive SARS-CoV-2 PCR with or without having detectable IgG against SARS-CoV-2. The mortality rate was higher in this subgroup compared with the other isolated subgroups (pneumonia only or MIS-C only patients). Based on the available literature, patients in this category tend to be older than those with MIS-C only features, and they more commonly have comorbidities [23].

Although in our patient, the diagnoses of pneumonia and MIS-C were both made on the day of admission, we cannot rule out the earlier initiation of viral infection as the proband’s father had some suspected signs of SARS-CoV-2 infection but was negative at the time of patient admission. The clinical characteristics and immunological profile in our case suggest some clues to differentiate type I IFN defects from common MIS-C cases [56, 57]. Age below 5 years, presentation of typical KD with short-term onset, dual manifestation with acute pulmonary infection, absence of leukopenia and thrombocytopenia, and lack of specific antibody response despite normal adaptive immunity could be helpful for differential diagnosis. In addition, the cytokine and autoantibody profiles identified in the proband appeared distinct from that of previously investigated MIS-C patients, although only a limited number of MIS-C patients have been studied thus far and no clear pathogenic autoantibodies have been identified [18]. Additional genetic variants or environmental exposures might be involved in the mixed presentations of immunodeficiency and immune dysregulation after COVID-19 infection in our patient. Our observation raises the possibility that inborn errors of, and/or dysregulation of type I IFN immunity, may potentially underlie a larger group of patients with MIS-C.

## Supplementary Information

Below is the link to the electronic supplementary material.

Supplementary file1 (DOCX 1499 KB)

## Data Availability

The raw data supporting the conclusions of this article will be made available by the authors, without undue reservation, to any qualified researcher.
