# Supplementary material for: Inherited IFNAR1 Deficiency in a Child with Both Critical COVID-19 Pneumonia and Multisystem Inflammatory Syndrome
Source: J Clin Immunol. 2022 Jan 28;42(3):471–83. doi: 10.1007/s10875-022-01215-7 (PMC8798309; doi:10.1007/s10875-022-01215-7)
Supplement: Supplementary file 1 — Supplementary file1 (DOCX 1499 KB) [file 10875_2022_1215_MOESM1_ESM.docx]

**Supplementary Data**

**Inherited IFNAR1 deficiency in a child with both critical COVID-19 pneumonia and multisystem inflammatory syndrome**

Abolhassani et al.

**Table S1-** WHO diagnostic criteria for multisystem inflammatory syndrome in children and adolescents temporally related to COVID-19.

| **MIS-C criteria** |
| --- |
| Children and adolescents 0–19 years of age |
| **AND** Fever > 3 days |
| **AND** two of the following:  1. Rash or bilateral non-purulent conjunctivitis or muco-cutaneous inflammation signs (oral, hands or feet).  2. Hypotension or shock.  3. Features of myocardial dysfunction, pericarditis, valvulitis, or coronary abnormalities (including ECHO findings or elevated Troponin/NT-proBNP),  4. Evidence of coagulopathy (by PT, PTT, elevated d-Dimers).  5. Acute gastrointestinal problems (diarrhoea, vomiting, or abdominal pain). |
| **AND**  Elevated markers of inflammation (such as ESR, C-reactive protein, or procalcitonin). |
| **AND**  No other obvious microbial cause of inflammation, including bacterial sepsis, staphylococcal or streptococcal shock syndromes. |
| **AND**  Evidence of COVID-19 (RT-PCR, antigen test or serology positive), or likely contact with patients with COVID-19. |

**Table S2-** Other rare variants in known IEI genes identified in the index patient.

| **Chr** | **Position** | **Zyg** | **Ref** | **Alt** | **Impact** | **Gene** | **EXON** | **mRNA change** | **Amino acid change** | **MAF** | **CADD_ score** |
| --- | --- | --- | --- | --- | --- | --- | --- | --- | --- | --- | --- |
| chr1 | 151789185 | het | G | A | MODERATE | *RORC* | 4//11 | c.253C>T | p.His85Tyr | 0.0004 | 27.3 |
| chr1 | 173916690 | het | G | A | HIGH | *RC3H1* | 14//19 | c.2554C>T | p.Arg852Ter | Novel | 35 |
| chr2 | 32461310 | het | T | C | LOW | *NLRC4* | 6//7 | c.619+6A>G | - | Novel | . |
| chr3 | 186944313 | het | G | C | LOW | *MASP1* | 11//15 | c.1442-5C>G | - | 0.0018 | . |
| chr7 | 98988511 | het | G | T | LOW | *ARPC1B* | 5//9 | c.501-5G>T | - | 0.0012 | . |
| chr12 | 49420515 | het | C | A | MODERATE | *KMT2D* | 48//54 | c.15234G>T | p.Met5078Ile | Novel | 18.11 |
| chr14 | 50121715 | het | G | A | LOW | *POLE2* | 12//18 | c.1018+8C>T | - | 0.0014 | . |
| chr14 | 106091714 | het | A | G | MODERATE | *IGHG4* | 2//4 | c.298T>C | p.Ser100Pro | Novel | . |
| chr16 | 50733809 | het | G | A | MODERATE | *NOD2* | 2//12 | c.484G>A | p.Val162Ile | 0.0002 | 1.421 |
| chr17 | 73832719 | het | C | T | MODERATE | *UNC13D* | 14//32 | c.1232G>A | p.Arg411Gln | 0.0008 | 23.1 |
| chr19 | 10463599 | hom | C | T | LOW | *TYK2* | 20//22 | c.3200+3G>A | - | 0.0014 | . |
| Chr: chromosome; Zyg: zygosity; Ref: reference allele; Alt: alteration variant; MAF: minor allele frequency in gnomAD database (https://gnomad.broadinstitute.org/); CADD: Combined Annotation Dependent Depletion score. | | | | | | | | | | | |

**Table S3-** Other rare homozygous variants in non-immune genes identified in the index patient.

| **Chr** | **Position** | **Zyg** | **Ref** | **Alt** | **Impact** | **Gene** | **EXON** | **mRNA change** | **Amino acid change** | **MAF** | **CADD score** |  |
| --- | --- | --- | --- | --- | --- | --- | --- | --- | --- | --- | --- | --- |
| chr2 | 111411041 | hom | CCAT | C | MODERATE | *BUB1* | 17//25 | c.1933_1935del | p.Met645del | - | . |  |
| chr3 | 129302411 | hom | G | A | MODERATE | *PLXND1* | 8//36 | c.2240C>T | p.Thr747Met | - | 12.79 |  |
| chr7 | 21934389 | hom | G | A | MODERATE | *DNAH11* | 79//83 | c.12920G>A | p.Arg4307His | 0.0004 | 23.2 |  |
| chr8 | 101270834 | hom | GGTT | G | MODERATE | *RNF19A* | 10//10 | c.2464_2466del | p.Asn822del | - | . |  |
| chr10 | 47911587 | hom | A | ACC | HIGH | *FAM21B* | 12//27 | c.1037_1038insCC | p.Gly347LeufsTer54 | - | . |  |
| chr12 | 7342352 | hom | C | T | LOW | *PEX5* | 1//14 | c.-17+6C>T | - | - | . |  |
| chr12 | 52865940 | hom | C | A | MODERATE | *KRT6C* | 2//9 | c.665G>T | p.Arg222Met | 0.0072 | 25.7 |  |
| chr13 | 42733465 | hom | C | T | MODERATE | *DGKH* | 6//29 | c.686C>T | p.Thr229Ile |  |  |  |
| chr15 | 23407347 | hom | C | T | MODERATE | *RP11-467N20.5* | 8//9 | c.1489G>A | p.Glu497Lys |  |  |  |
| Chr: chromosome; Zyg: zygosity; Ref: reference allele; Alt: alteration variant; MAF: minor allele frequency in gnomAD database (https://gnomad.broadinstitute.org/); CADD: Combined Annotation Dependent Depletion score. | | | | | | | | | | | | |

**Table S4-** Primers used for confirmation of the large deletion in the *IFNRA1* gene.

| **Targeted region** | **Sequences** | **F/R** | **Tm** |
| --- | --- | --- | --- |
| Exon 7-8 | 5’_ CCCTAACATAGTGTCTGGCAAT_3’ | Forward | 60^o^C |
| Exon 7-8 | 5’_ CATTCAAGCTTTATTTACCCAAA_3’ | Reverse | 60^o^C |
| Exon 6 | 5’_ GCGAGCCTTTATCTTCTTGC_3’ | Forward | 60^o^C |
| Exon 6 | 5’_ GGGCAAAAGGAAATTTATGGA_3’ | Reverse | 60^o^C |
| Exon 9 | 5’_ TTCCCTGATTTCTTGAGGTGA_3’ | Forward | 60^o^C |
| Exon 9 | 5’_ CGACTTGAGAGTTAAGCGTTACAA_3’ | Reverse | 60^o^C |
| Intron 6 | 5’_ GAGACCCCCATCTCTAAGCA_3’ | Forward | 60^o^C |
| Intron 8 | 5’_ CAGCCTGGGAAAAAGAATGA_3’ | Reverse | 60^o^C |

**Table S5-** Localization of large deletion of the *IFNRA1* gene in the index patient.

| **Forward Primer Designed for intron 6 of *IFNRA1* gene** |
| --- |
| 5’_ GAGACCCCCATCTCTAAGCA_3’ |
| **Intron 6 remained in the proband** |
| 5’_TTAAAATTTTTAAAAAGCTGATAAAAGAGAACTGCATGTAGTTTATATTGATTGGAAAATTGTAGGAGATGAATAGAGAAAAGAGAAGAGGAAAATTAAGTAGAAGGAAAAATGGGTTATGGGGAGAAAAGAGATGGAAAGAAAGATATACATTGGTGGCAGAGGAGTAGAGAGAGCAAAGGCAAGTATAGCTAAGACAGGAAAGGAAAAGTCAGAAGAAACTGGAAAGAAACAGAAAAAATGAGGGAGAAGGGAAGTGTAAGTACCGCTATGATCAATTTCTATCTTTTAGTGTTAATTTTGAACCAAGGGTGACTGGTAGAATGCAAATGCATTTATGTGCATATGTGCCCCACAATGCATATATAACCTCACAAACCAATCAGGAAACTGTTCAGTAGTCTTTTGTGTATACCTCACTTTCAGAAGTGAATAAATAATAGGGTCACCAAATAGATAATGAGAGGTATTTCTGATAGCTGATCTCTAAGCTTCTTTTGGGTACTTACCATATTGTACAAAGCACTGAAGAAATGGAAAGAGTCAGCAGAATCAAACGTGGTTCAAAAATTGAAAGCAGTTAGCCAACATTGCAAAGTGTTGTGTGGTTTAGGACTAAAGAGTGGTATACAAATTATAAGGGTTGCAGAAATTGAAAATGTAAAGGTGAATAGGGGCTAAAGAGACCAAAGAAGCCATAAGGAAAGAGATAAGGTATGAACAAGGCCTTGATTGTTAATTCAGATTTTTTTTAAGTAGACAACTATTAGATGTTAGTTTTCTGTGTCTGTAACTAATTACCACAGACTTAGCAACTTAAACAGCTGTTTATTGTCTCAAAGTTTCTGTGAGTCAGGAGTCCAAGCGTGGCTTAGCTGGCTCTTCTGCTAAGCAGT_3’ |
| **The shared sequence between intron 6 and intron 8** |
| 5’_ CAAGGAG_3’ |
| **Deleted sequence from intron 6-exon7-intron7-exon 8- intron 8 in the proband (4394 nucleotides)** |
| **5’_TCAGTCATTCATACAAGACTTGGGGGCCGCTCCCAAGCTCACATGTTGTTGGCAGAATTTATTTCTAGAGCTCATGGTGGCTCACTTCTTAGAGGCCATCAGGAAGGGAGAGTCTGACCTCTAGATCCTTTTTTTTTTTTTTTTGAGACGGAGTCTCACTCTGTCACCCAGACTGGAGTGCAGTGACATGATCTCAGCTCACTGCAACCTCCGCGTCCTGGGTTCAAGTGATTCTTCTGCCTCAGCCTCCCAAGTAGCTGGGACTACAGGTGTGCACCACCACACCCAGCTAATTTTTGTAGTTTTAGTAGAAACGGGGTTTCACCGTATTGGCCAGGCTGGTCTCGAACTCCTGACTTCAGGTGATCCACCCGCCTCAGCCTCCCAAAGTTCTGGGATTATAGGCGTGAGGCACCGCGCTCAGCCATTTTTTTTGTATTTTTAGTAGAGACGGAGTTTCTCCATGTTTGCCAGGCTGTTCGCAAACTCCTGGCCCCAAGTGATCTGCCCACCTCAGCCTCCCAAAGTGCTGGGATTAGAGGCATGAGCCACCATGCCTGGCCTAGATCCTCTTTTAAGGGGTTCATCTGATTAGGTCAGGCCCACCCAGAATAGTCTCTCATTTGATTCAACTGATTTGGGACCTTAATTACATCTGTGAAATACCTTCTCTTCTGCCACTTAATGTAACCTAATTATGAGAGGAGGGCCTCCATCGTATTTACAGGTCCTGCCCATGCTCAAGAGGAGGGGATTATACAGGATGTGTACACCAGGGGGCAGGAATCTTGGGGGCCATCTCAGAATTTTGCCTTCTCCAGTGGGGAGGATGTCCTTCTAGAGTAGCAGAACATTTGGACACCAGGTGGCAATGGGAAGAAAGCTGAATAAGTAGAATGGGACAAGATTAGGGTCAACCTCAGGCTGTCCTGTAGGCTTTTGGGGATTTTGTGTTGGGGTGATAGTTAACCTGAGCTCTGGATTTGAGGAAGGTGCATGCGGCGACATGGTATGTGGGAGAGTGGACTAAGGAGACACTAAAGAGAGATAAGGAAACCAGTCAGGAGGCTATTAGAGTTCTGGGCTTGAACTGAGATGCTTTTAGCTGTTGGTTTCTGGGCAAGTTACTTAACCTCTTTAGTCTCGTACTTATACTTGCTCTTATAACCCCGCAGTGTGTCTCTATTTTAGCACTTAATCAGTGTCCATTGGTATGCTGGCGCATGTTTAGCAGCTGGCTTTCCAGAAAGAAAAAGTCCTATTTTGTAGTGTTTGCTGATTTTTATGATATAAATATTCCCACCATGGCCACTTTCAAGCCACCAGCCTGATGTCCCTGAATATGGAGTTGGGAACATATGCACAGTAGCACACCACTATCATATAGCTACAATAGATAAAGATAGCCCCAAGAGCTTAGAAAATAAAATGTAGTAAAATAATTAGGACAAGATGAATTTATTTATTACCTCTGTCTTTAAAACAATTTTTTATTTGCAAATTTATATAACTTAATTTTTAGTAATGGTTTTTCTTTAAAAACCAGTTTGTATTTTAGGAGGCCGAGGTAGGTGGATCACCTGAGGTCAGGAGTTCAAGACCAGCCTGGCCAACATGACGAAACCCCATCTCTACTAAAAATACAAAAATTAGGCAGGCCTGGTGGCGTGCACCTGTAATCCCAGCTACTCAGGGGGACAGAGGCAGGAGAATTGCTTGAACCCAGGAGGCAGAGGTTGCAGTGAGCCAAGATCCTACCACTCCACTCCAGCCTGGGTGACAGAGCGAGACTCGGTCTCAAAAAAAAAGCCAAAAAAAATAAACCAGCTTGCAGCATTCCTGGAAATTCTAACTAACAGATGTTCTTGCATATTGATATGAGCCACCTCCAGCAGAGCACAACATGACCACAGTCTGGAACAGTCTTTGGTTTTCTTTTATGTTAGATGCATATCTCTTCCATTGTTTGTGAGTTTCCTGAGTGTGGATACTATTTATTTCTGTAACCTTAGCCCCTAACATAGTGTCTGGCAATTGTAAATACTTAATAAATATCTAATGAATTTAAAAAATATTTGTCTTAAAAGCGCCTTTTTAAAAAGGAATCCTGGAAACCATTTGTATAAATGGAAACAAATACCTGACTGTGAAAATGTCAAAACTACCCAGTGTGTCTTTCCTCAAAACGTTTTCCAAAAAGGAATTTACCTTCTCCGCGTACAAGCATCTGATGGAAATAACACATCTTTTTGGTCTGAAGAGATAAAGTTTGATACTGAAATACAAGGTAAGGCAGTAGTTTTTACTGGAGATTGTAATTCTCTGGTGCAAGTTTTTAAAATTGTTTTTCTAATTGAACATTATTTCTTTACAAATTTTTTCTAGCTTTCCTACTTCCTCCAGTCTTTAACATTAGATCCCTTAGTGATTCATTCCATATCTATATCGGTGCTCCAAAACAGTCTGGAAACACGCCTGTGATCCAGGATTATCCACTGATTTATGAAATTATTTTTTGGGAAAACACTTCAAATGCTGAGGTAAAAAGACTGTATAGTATAATTTTGTAACTTAGAGTTATAATTATGATTTGGGTAAATAAAGCTTGAATGTAAAATTTGGGGGAAATTTTTAAACTTTATGTGGGCTGGATGCAGTGGCCTGTAATCCCAGCACTTCAGGAGGCCAAGGCGAGAGGATCACTTGAGCCTAAGGGTTTGAGACCAGCCTGGGCAACATAGGGAGACCCTGTCTCAATAAAAATTTTAAAAAATTAGCCTGGTGTGGTGGCGTGCACCTATATTCCCAGCTACTTGGGGTGGGAGGGTCACTTGAACCTGGGAGGTCAAGGCTGCAGTGAGCCATGATCGTGCCACTGCACTCCAGCCTGGGTGACAGAGTGACTGACAGCTTGTCTTTAAAAAAAAAAAATGTGATTAACTCAGATATTAACAAAATGAAGATTATGAGCATTTTTCATGTTTTGCACTGTAGAGTTATGGGTGAGCTGCATCTGGGCCCCAGTTTGCTTTTAAAATACAGATTCCTGAGCCTATTGTTACTGAATCATTATCTCTGGGGATGGAACTCAGGAATTTGCATTTTTAACATGATTCCCATGTATTCATCTAAACCTGGGATTCTCAGCACTATTGACGTTTGGGCTGGATAGTTCCTTGGGAGGGGGCTGTCCAGTGCAGTGCAGGATGCTTGGCAACATTCTGGTTTCTGCCTACTAGATGCCAGTAGTGTTCCTTCCTAGTTGTGACAACCAAAAACATCTTCAGATATTGTCAAATGTCCCCTGGTGGCTCCTCCAAGGGGACAAAAATTGGTTCTTATGGGACAAAAGAATTTATGTAGTACAGTTGTTTGTTTTCCTCCAAAACCATTGGAAAGCATTCTTCCAAAGTTCAGCTTTGCCCAACAAAATCTTATTCCTTAGTATTTAATTTTATGATGGGGGAAGGATTAGGAAAAAATTGCCCAAAAAGTTGTTTAGTTGGGGAGGTAATGAAAAAAGGGTTGACAAACACTGGTCTAAACCCTAAAATTCAGATACCACTGACAAAGATAATATATACCGTCAGATAATATACGTGTTTCCCAGATCACTTCCCCCTCGTGGACTTGTAGTCAAGAATCGACCTTTAAAACTCCTGTCCAGTCAGGCTGGTAAATTCTTTAACTTCACCTCATTTTCTATTGGAAATCTAAGCAAATTCCATTGATTTGGCTGCTTCCCTTTTTA** |
| **ATTGTCTGACAACCCTGTAACCATAGCTTAATGTAGCCCATTGAGAATTATGGTCGGTTTTCAGATGTCTGTAGATCAGACAAACCTAAATTTCTATCTCACTGCTAAACTGTGTAACCTTACGTACGCAGGGTGCTTTACCTCTTGGAGACTTTGCTTCCTCTTCTCTGAAGTGGTGGTAATCATAACAACTAAATTTTTCAAAAGGATGTTTATAAGGTTTAAAAGGAAGTAACAAATATGAAAATACCTAGCACATTGCTGGACAAGTAGTAGAGATTCAATAAATTGTAGTTGCCATCTTAACCTATACTGGATAATATATCAATAATTGTTACTATAGAAACTCTTATTGAATACTTATATGTAAGGTACTATGCTAAGTATTGTACATGAATTGTCTCAGTCATTCTTCAAAACAACCCTGCCGGATTATGTGATTGGTTACATTGCTGTTTGGCAAATACTTGGGACGGTGACTTCTTCCCTGCACCACCAGACCGTAGATGCTGGAGCCCAAGTAGATGTGCTGAGAACAGAGGCCTTAAGTGTGCTTGCAGGGTTTGGCTTGGCTCTTGCACTGCTCCTATTTGCCAGGAATAAAATGTATTCCACATAGTCTCTGGTC_3’** |
| **The shared sequence between intron 6 and intron 8** |
| 5’_ CAAGGAG_3’ |
| **Intron 6 remained in the proband** |
| 5’_GATTAGAGGCTGCTGGAGCTGACCTAAACCCCATGCACAGCCTCCGTGGGAGCTGCCCAGCCAGTCTGTAGACTTGTGAGTGAGAAACTACATAATCGTTTTGTGAGCCACTGAACGTTAAGGCAGTTTTGTTATCTATATTTTTTTATTTTATTTTATTTTATTTTTTTTTTTTTTGAGACAGGTTC_3’ |
| **Reverse Primer Designed for intron 8 of the *IFNRA1* gene** |
| 5’_TCATTCTTTTTCCCAGGCTG_3’ |

**Table S6-** Plasma sample of index patient was screened for autoantibodies against multiple interferons using a bead-based protein array.

| **Parameters** | **Index patient (MFI)** | **Mean normal population (MFI)** |
| --- | --- | --- |
| IFNA21 | 69 | 126.9 |
| IFNA5 | 44 | 69.6 |
| IFNA4 | 118 | 102.8 |
| IFNW1 | 66 | 46.4 |
| IFNA6 | 384.5 | 439.2 |
| IFNA10 | 86 | 94.7 |
| IFNA8 | 327 | 349.1 |
| IFNA4*1 | 121 | 108.9 |
| IFNA17 | 50.5 | 66.7 |
| IFNA Alpha | 80 | 83.4 |
| IFNA2 | 198 | 160.9 |
| IFNA1 | 57.5 | 86.3 |
| IFNG | 47 | 57.4 |
| IFNB1 | 34 | 36.7 |
| GAD2 | 59 | 62.7 |
| IL22 | 58 | 77.3 |
| IL17F | 1097.5 | 320.1 |
| AADC | 84 | 108.3 |
| TGM2 | 45 | 149.7 |
| TGM3 | 100 | 297.6 |
| PVALB | 69 | 80.8 |
| TGM4 | 44 | 45.5 |
| CXCL10 | 45 | 66.7 |
| MSRA | 44 | 58 |
| Anti-IgG | 20712 | 18165.2 |
| EBNA1 | 19017 | 18071.6 |

| **Table S7-** Plasma sample the index patient was screened for autoantibodies against 9000 proteins. Top 50 auto-antibodies target proteins with higher ratio in the patient compared to healthy controls have been listed. | | | |
| --- | --- | --- | --- |
| **Description** | **Index patient** | **Average healthy controls** | **Ratio index patient to healthy** |
| kallikrein-related peptidase 7 (KLK7), transcript variant 1 | 967 | 24.5 | 39.4 |
| hypothetical protein FLJ22795 (FLJ22795) | 39 | 2 | 19.5 |
| plasminogen activator, urokinase receptor (PLAUR), transcript variant 1 | 201 | 20.5 | 9.8 |
| Kelch-like ECH-associated protein 1 | 1133 | 121 | 9.3 |
| Kelch-like ECH-associated protein 1 | 1223 | 134 | 9.1 |
| anthrax toxin receptor 1 (ANTXR1), transcript variant 3 | 8 | 1 | 8.0 |
| Huntingtin interacting protein K (HYPK) | 158 | 20 | 7.9 |
| coiled-coil domain containing 32 (CCDC32), transcript variant 2 | 29 | 4 | 7.25 |
| Huntingtin interacting protein K (HYPK) | 172 | 24 | 7.1 |
| protein O-fucosyltransferase 2 (POFUT2), transcript variant 3 | 12 | 2 | 6.0 |
| unkempt homolog (Drosophila)-like (UNKL) | 586 | 98 | 5.9 |
| unkempt homolog (Drosophila)-like (UNKL) | 586 | 99.5 | 5.8 |
| NADPH oxidase activator 1 (NOXA1), mRNA | 143 | 26 | 5.5 |
| mitogen-activated protein kinase 8 (MAPK8), transcript variant JNK1-a1 | 21 | 4 | 5.25 |
| LIM and senescent cell antigen-like-containing domain protein 1 | 1706 | 326 | 5.2 |
| LIM and senescent cell antigen-like-containing domain protein 1 | 1632 | 323.5 | 5.0 |
| S100A1 Protein | 9038 | 1797.5 | 5.0 |
| NADPH oxidase activator 1 (NOXA1), mRNA | 143 | 29 | 4.9 |
| myeloperoxidase_IV | 162 | 34 | 4.7 |
| Pleckstrin homology domain-containing family O member 2 | 184 | 39 | 4.7 |
| Pleckstrin homology domain-containing family O member 2 | 164 | 39 | 4.2 |
| protein phosphatase 1, regulatory (inhibitor) subunit 8 (PPP1R8), transcript variant 3 | 133 | 33 | 4.0 |
| 6-phosphofructo-2-kinase/fructose-2,6-biphosphatase 1 | 238 | 59.5 | 4.0 |
| chromosome 2 open reading frame 34 (C2orf34) | -4 | -1 | 4.0 |
| dsDNA_plasmid | 8 | 2 | 4.0 |
| Dual specificity mitogen-activated protein kinase kinase 6 | -4 | -1 | 4.0 |
| Golgi-associated plant pathogenesis-related protein 1 | 4 | 1 | 4.0 |
| mitogen-activated protein kinase-activated protein kinase 3 (MAPKAPK3) | 12 | 3 | 4.0 |
| Paraspeckle component 1 | 4 | 1 | 4.0 |
| PD1 / PDCD1 Protein | 4 | 1 | 4.0 |
| polymerase (RNA) III (DNA directed) polypeptide F, 39 kDa (POLR3F) | 4 | 1 | 4.0 |
| protein-kinase, interferon-inducible double stranded RNA dependent inhibitor, repressor of (P58 repressor) (PRKRIR) | 4 | 1 | 4.0 |
| S100A9 / CAGB / p14 Protein (His Tag) | 4 | 1 | 4.0 |
| ssDNA | 16 | 4 | 4.0 |
| syntaxin 18 (STX18) | 4 | 1 | 4.0 |
| syntaxin 18 (STX18) | 4 | 1 | 4.0 |
| Syntaxin-10 | -4 | -1 | 4.0 |
| F-box protein 4 (FBXO4), transcript variant 2 | 78 | 20.5 | 3.8 |
| F-box protein 4 (FBXO4), transcript variant 2 | 76 | 20.5 | 3.7 |
| synuclein, beta (SNCB), transcript variant 2 | 33 | 9 | 3.6 |
| 6-phosphofructo-2-kinase/fructose-2,6-biphosphatase 1 | 205 | 57.5 | 3.5 |
| growth arrest-specific 7 (GAS7), transcript variant b | 21 | 6 | 3.5 |
| ring finger protein 1 (RING1) | 111 | 33 | 3.3 |
| ring finger protein 1 (RING1) | 111 | 33 | 3.3 |
| mitogen-activated protein kinase kinase kinase 14 | 90 | 27 | 3.3 |
| Fc receptor like 2, mRNA (cDNA clone MGC:71141 IMAGE:3529386), complete cds | 66 | 20 | 3.3 |
| chromatin modifying protein 2B (CHMP2B) | 162 | 53.5 | 3.0 |
| mitogen-activated protein kinase 8 (MAPK8), transcript variant JNK1-a1 | 12 | 4 | 3.0 |
| mitogen-activated protein kinase kinase kinase 14 | 86 | 29 | 2.9 |
| Jun B proto-oncogene (JUNB), mRNA | 53 | 18.5 | 2.8 |

**Figure S1-** Trends of complete blood counts (A) and inflammatory markers as well as viral load indicated with Ct values of PCR results (B) during the hospital admission of the index patient.

**
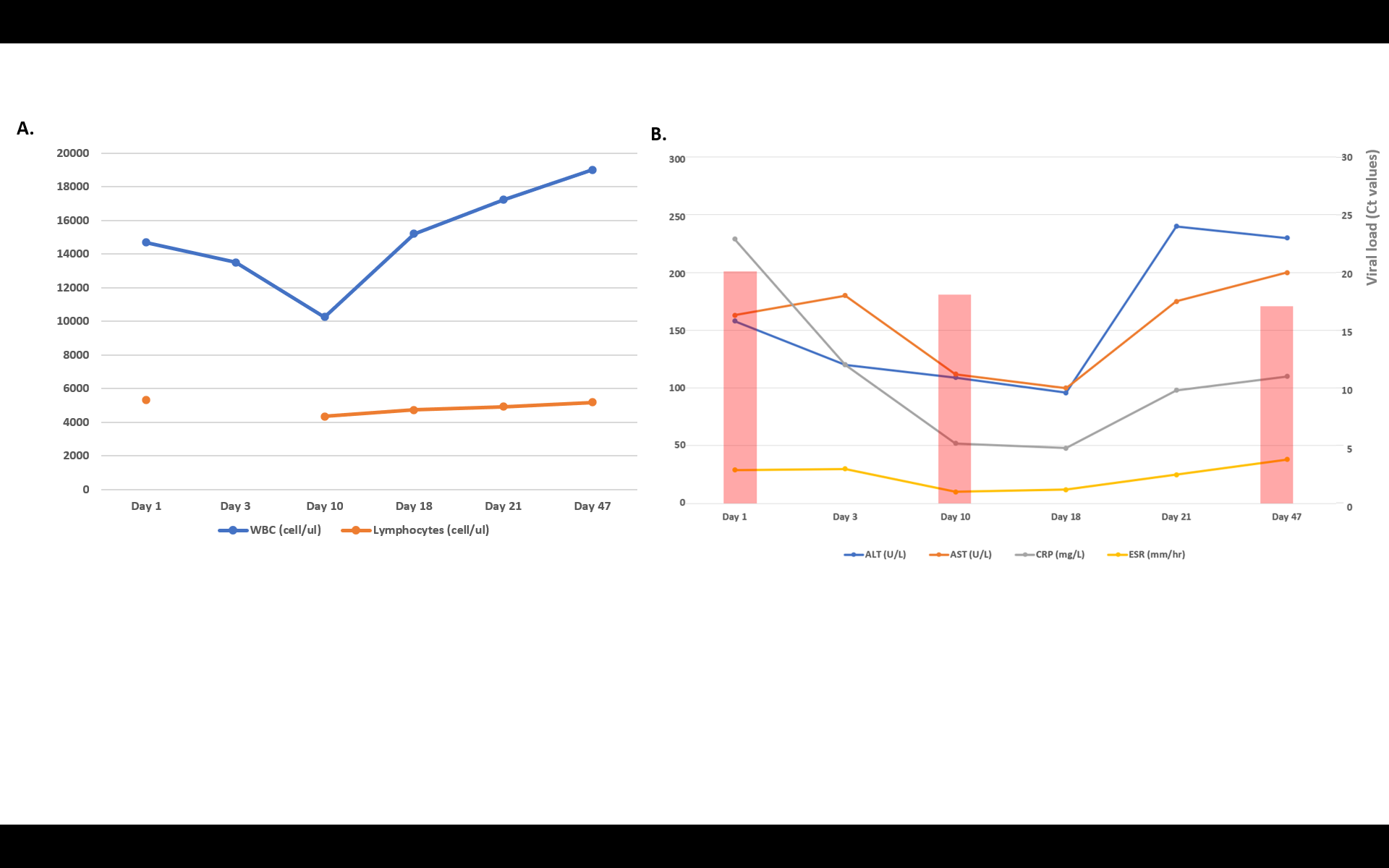

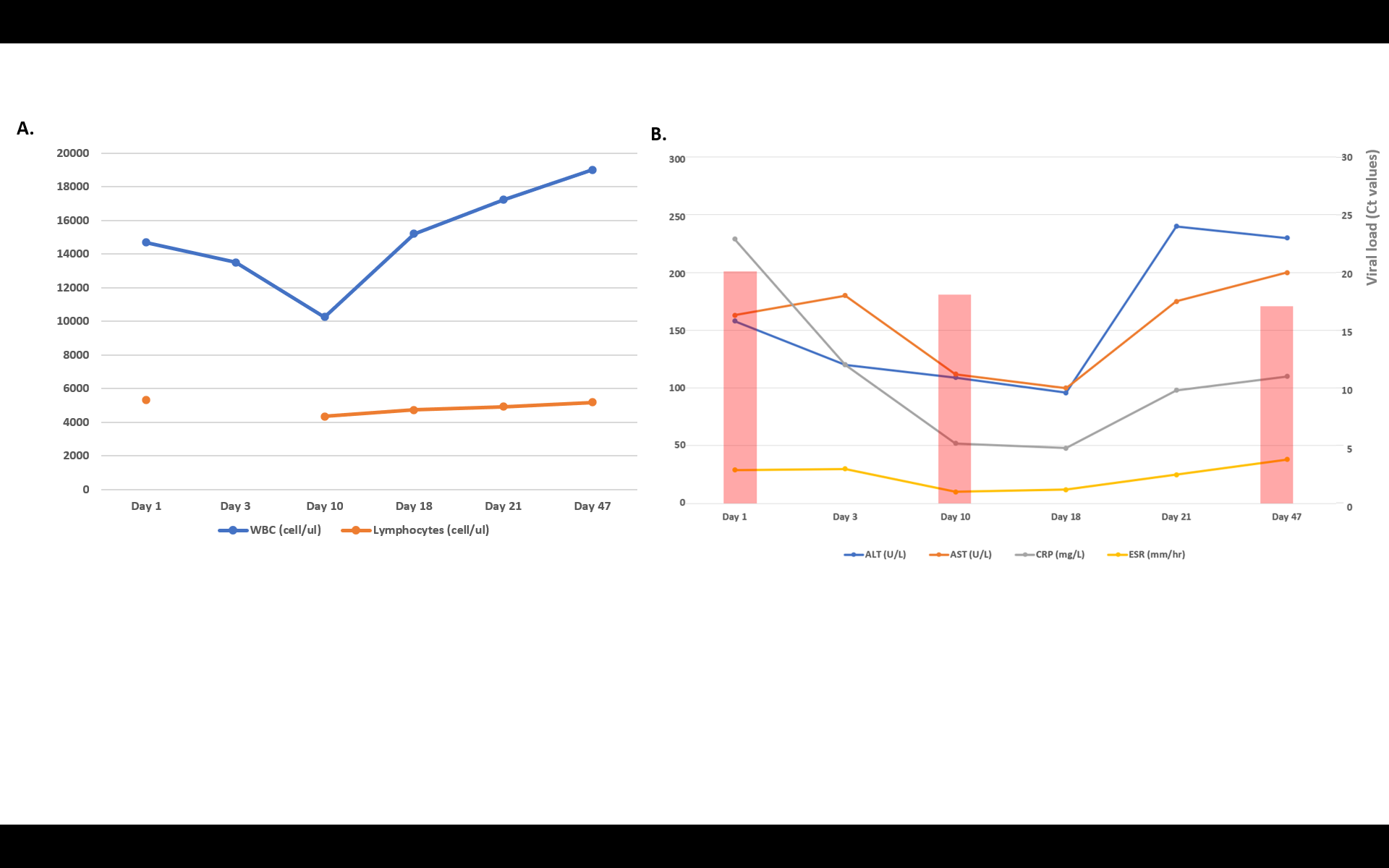
**

**Figure S2-** Complete sequences between intron 6 and intron 8 of the *IFNRA1* gene in a child with both critical COVID-19 pneumonia and MIS-C.


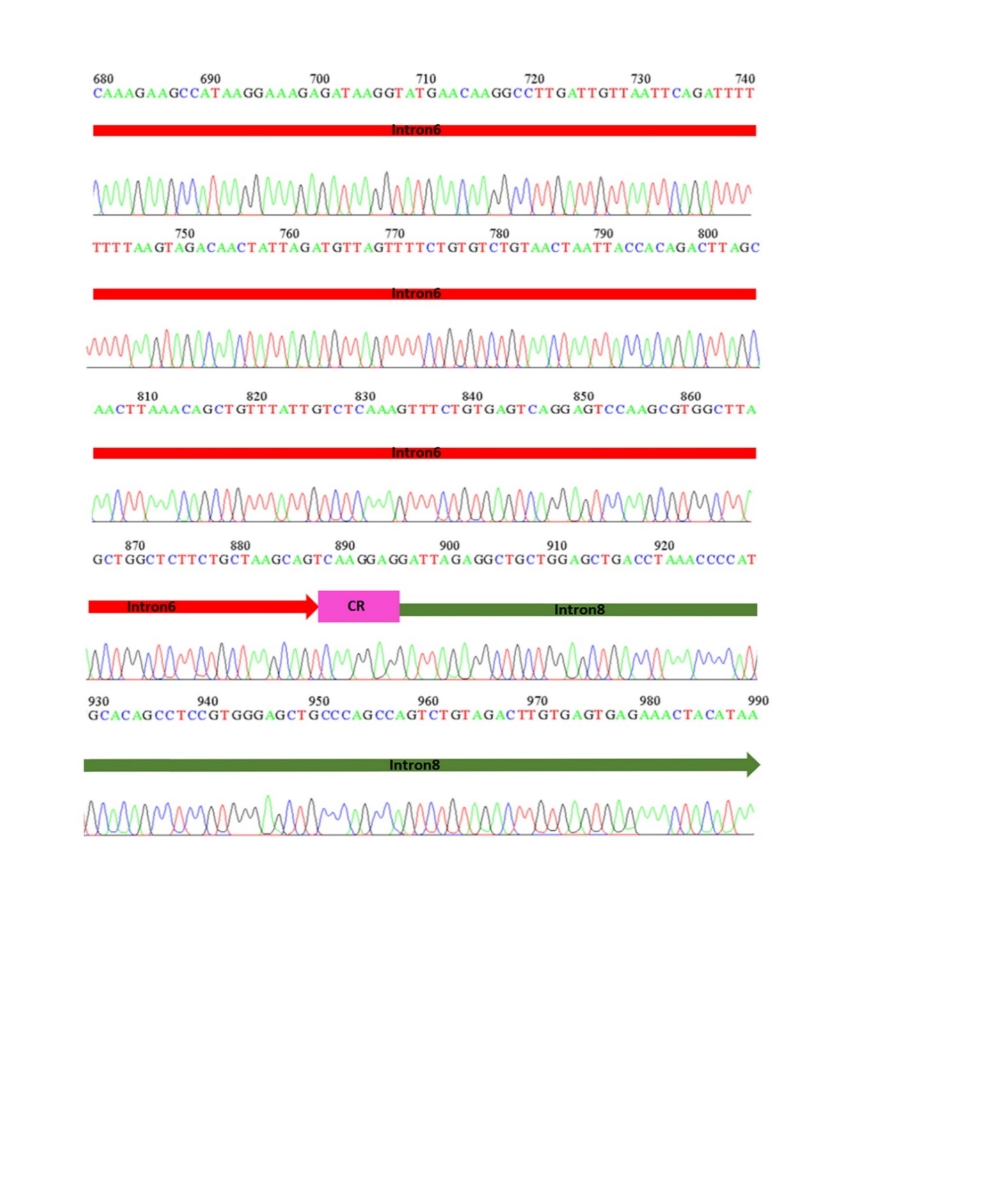


**Figure S3-** Transposon elements (TE) analysis in the large-deleted region in a child with both critical COVID-19 pneumonia and MIS-C.


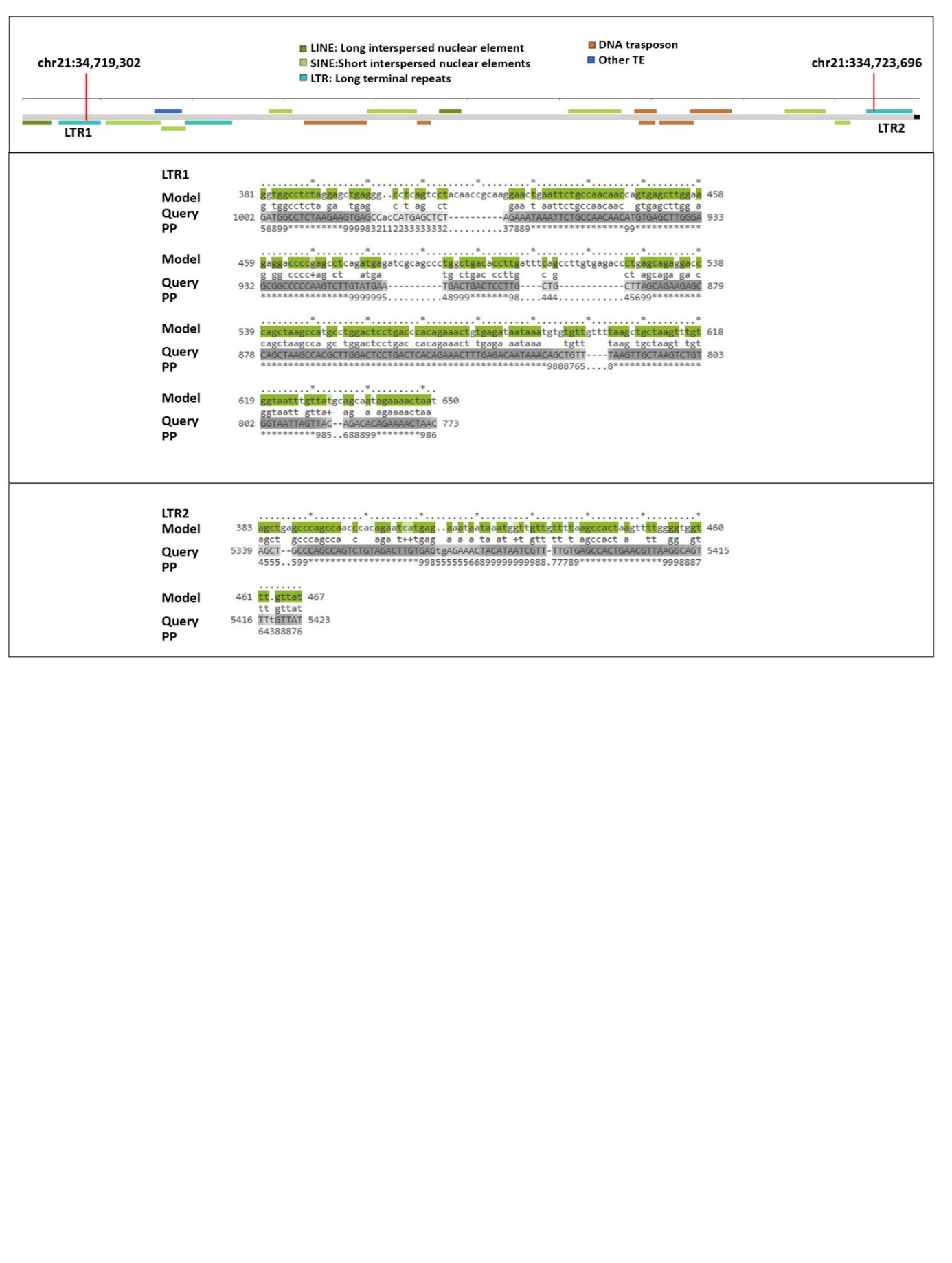


**Figure S4-** Specific IgG antibody profile of the index patient (IFNAR1-deficient). **Top,** Shown are heatmaps depicting adjusted species score values. Intravenous immunoglobulin (IVIG), human IgG-depleted serum (IgG-depleted) and mock-IP samples served as positive and negative controls, respectively. **Bottom**, Bar graph depicting the response factor for each sample, which is the sum of microbial species for which antibodies were detected (grey) or passed the significance threshold (blue) in a given sample, respectively, divided by the total number of microbial species for which antibody-peptide interactions were detected in any sample


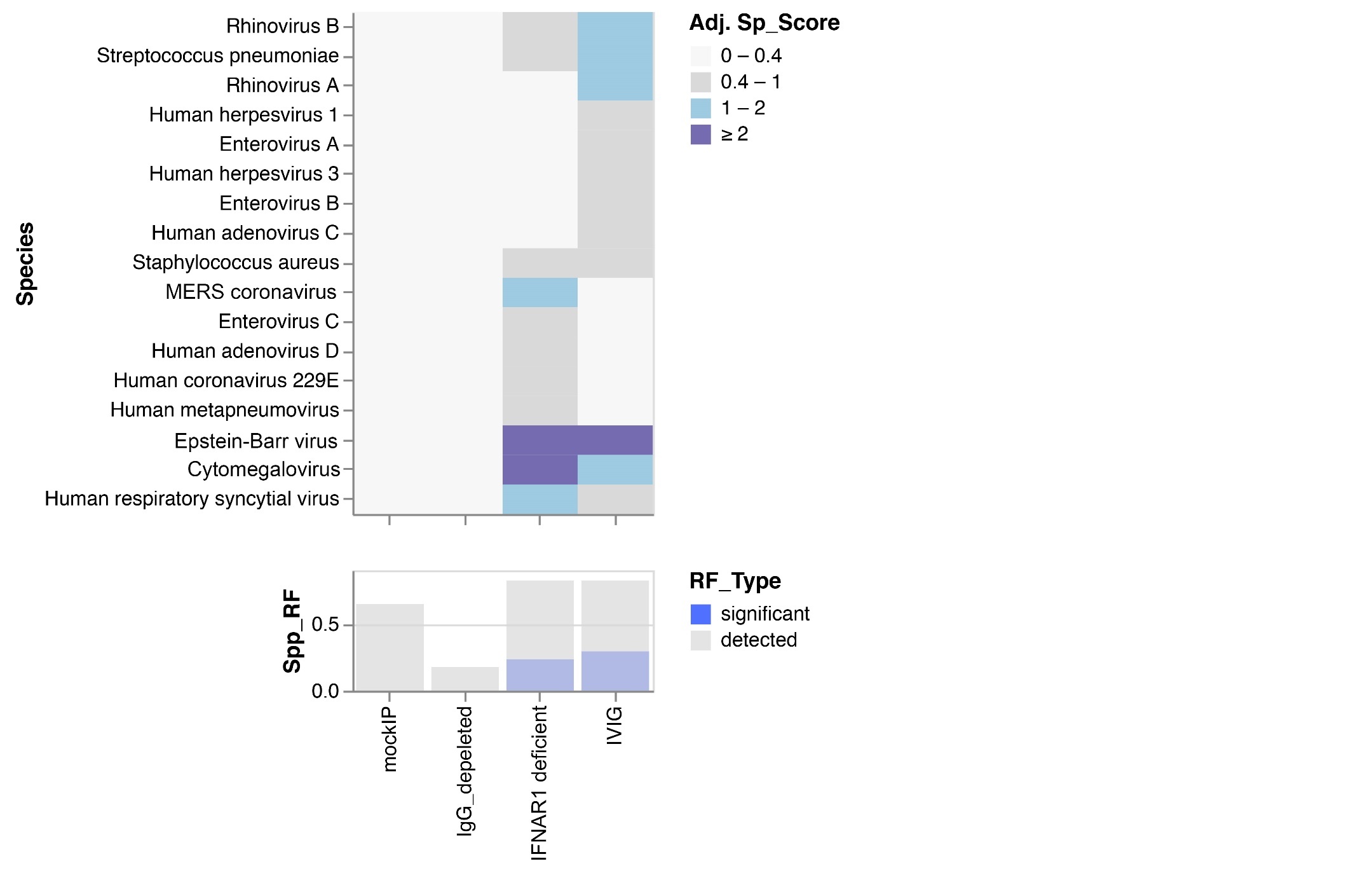


**Figure S5-** Serum levels of the indicated cytokines as measured by automated ELISA in the proband with *IFNAR1* mutation comparing with healthy controls, pediatric COVID-19, KD, MISC, mild adult COVID-19, severe adult COVID-19 and toxic shock syndrome patients (TSS) [39].


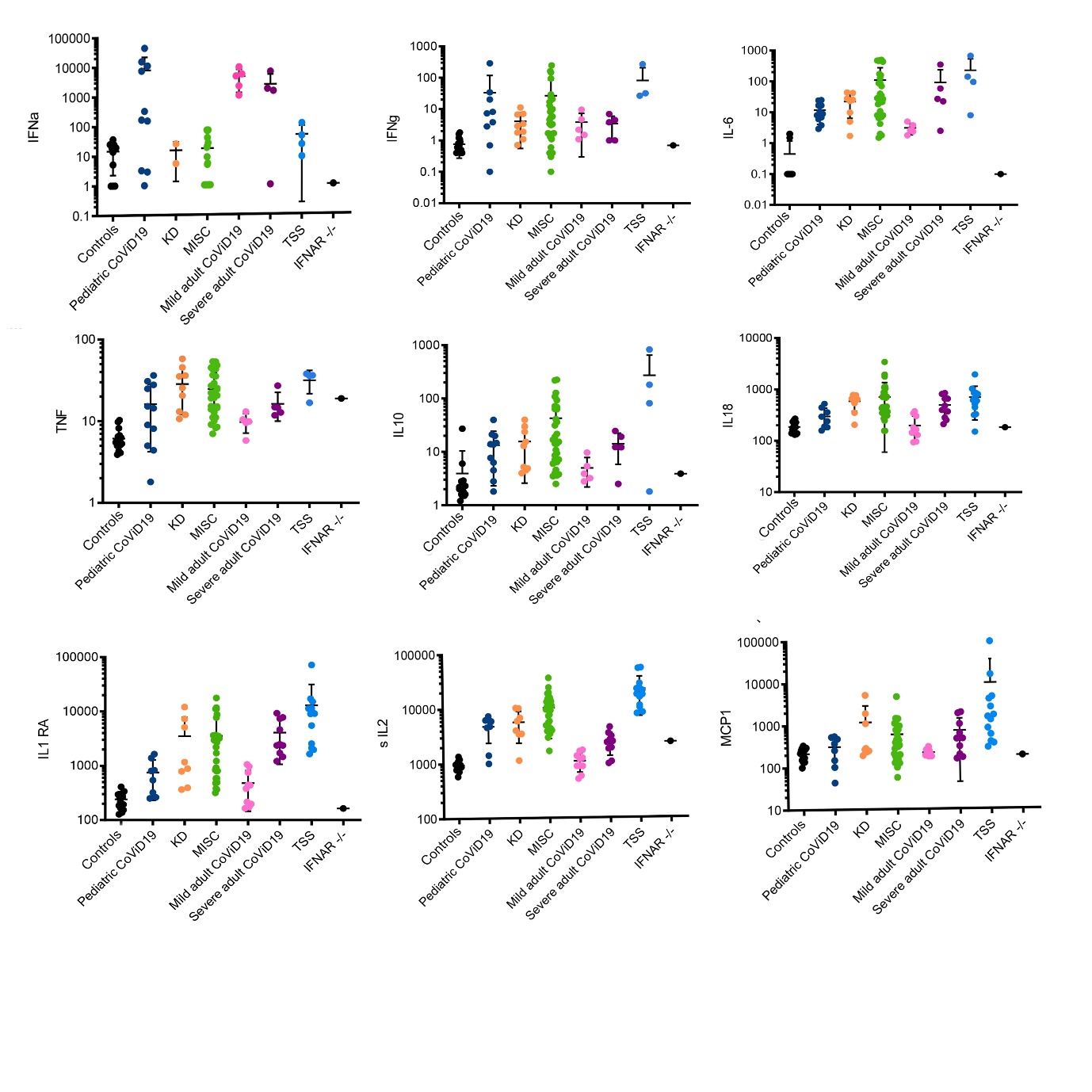


**Figure S6-** The patient with *IFNAR1* mutation was screened for autoantibodies against multiple interferons using a bead array (see Table S6). An APS-1 patient with *AIRE* mutation and with previously known autoantibody reactivity against type I interferons (IFN) was included as a positive control. Healthy blood donors (n=98) were also included in the same experiment. The patient with *IFNAR1* mutation was negative for all investigated type I IFNs, including the here shown IFNA2 (A) and IL17F (B). The upper limit of the normal range was defined as five standard deviations above the average of the healthy controls.


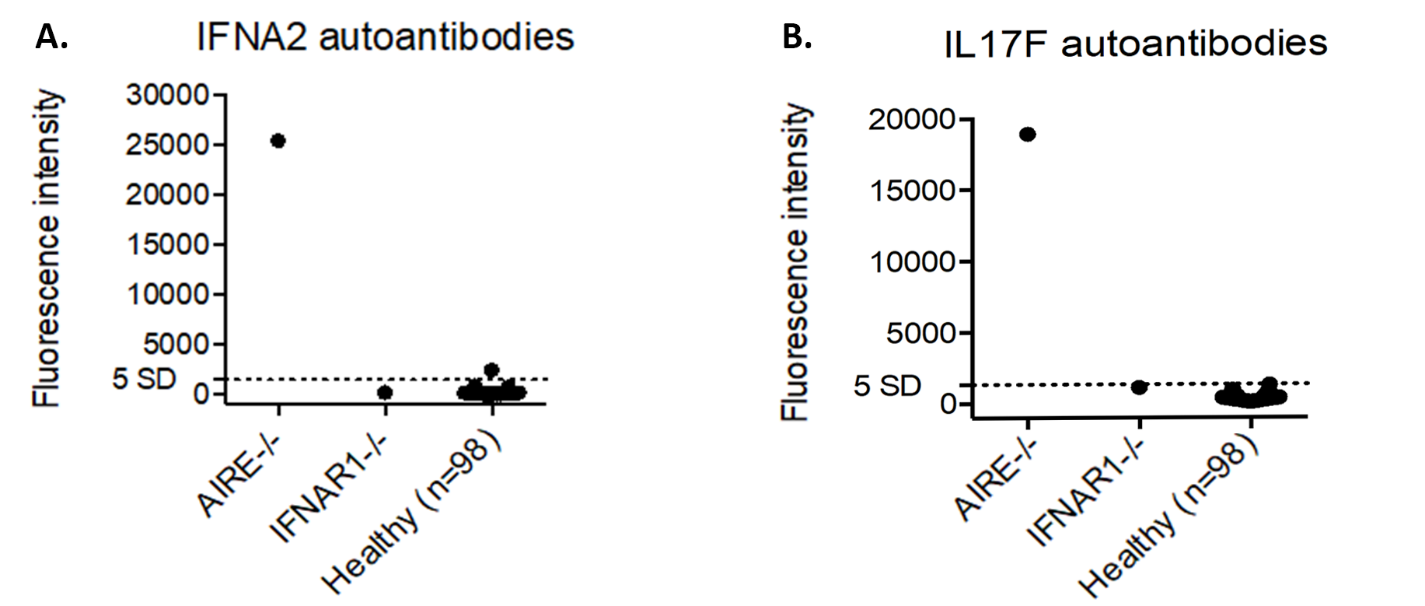


**Figure S7- Top hits from proteome-wide autoantibody screening.** The IFNAR1-deficient patient was screened for plasma autoantibody reactivity against a panel of around 9000 full-length human proteins. Two blood donors were included as controls. Proteins were ranked based on the signal intensity ratio between the IFNAR1-deficient patient and the average of the healthy controls. Only proteins with mean fluorescence intensities over 100 arbitrary units were included. Hits with more than five times elevated signal for the IFNAR1-deficient patient are shown.
